# Label-free quantification of calcium-sensor targeting to photoreceptor guanylate cyclase and rhodopsin kinase by backscattering interferometry

**DOI:** 10.1038/srep45515

**Published:** 2017-03-31

**Authors:** Stefan Sulmann, Amanda Kussrow, Darryl J. Bornhop, Karl-Wilhelm Koch

**Affiliations:** 1 Department of Neuroscience, Biochemistry group, University of Oldenburg, D-26111, Oldenburg, Germany; 2 Department of Chemistry, The Vanderbilt Institute for Chemical Biology, Vanderbilt University, Nashville, TN 37235, USA

## Abstract

Quantification of protein binding to membrane proteins is challenging and a limited set of methods is available to study such systems. Here we employed backscattering interferometry (BSI), a free-solution label-free method with high sensitivity, to quantify the interaction of neuronal Ca^2+^-Sensor proteins with their targets operating in phototransduction. We tested direct binding of guanylate cyclase–activating proteins (GCAP1 and GCAP2) to their membrane target guanylate cyclase 1. The regulatory mechanism of GCAPs including their binding interface in the target is unresolved. Here we used a label-free, free-solution assay method based on BSI to determine binding constants of GCAP1 and GCAP2 to the full-length membrane-bound guanylate cyclase type 1. GCAP1 and GCAP2 bound to different regions on the target guanylate cyclase with submicromolar affinity (apparent K_D_-values of 663 ± 121 nM and 231 ± 63 nM for Ca^2+^-free GCAP1 and GCAP2, respectively). A guanylate cyclase construct containing the juxta-membrane and kinase homology domain harbored an exclusive binding site for GCAP1 with similar affinities as the full-length protein, whereas GCAP2 did not bind to this region. We provide a model in which GCAP1 and GCAP2 do not share a single binding site to the target, thus cannot exchange upon fluctuating Ca^2+^ levels.

Photoreceptor cells efficiently respond to changing light conditions on a millisecond time scale by a well-balanced interplay of two second-messenger species, cGMP and calcium^1^,^2^,^3^. Light excitation of the G protein-coupled receptor rhodopsin triggers a downstream signaling cascade leading to hydrolysis of cGMP, subsequently leading to a decrease of cytoplasmic Ca^2+^-concentration [Ca^2+^]. A network of Ca^2+^-sensor proteins in rod and cone photoreceptor cells can detect subtle changes in intracellular [Ca^2+^]. These Ca^2+^-sensors precisely regulate the enzymatic activity of their targets^4^,^5^,^6^,^7^ providing an efficient Ca^2+^-mediated feedback loop to restore second messenger levels prior to illumination. Among them the guanylate cyclase-activating proteins (e.g. GCAP1 and GCAP2 in mammalians) control the synthesis of cGMP by sensory guanylate cyclases (GCs) in a Ca^2+^-dependent manner and in a step-by-step Ca^2+^-relay mechanism^4^,^6^. GCAPs contain three functional and one non-functional EF-Hand type of Ca^2+^-binding motifs and are Ca^2+^-sensor proteins belonging to the family of neuronal Ca^2+^-sensor (NCS) proteins^4^,^5^,^6^,^7^. Mammalians express two or three, while teleost fish express six to eight different GCAP isoforms in their rod ancd cone cells^8^, which all regulate enzymatic activity of sensory GCs at different Ca^2+^ levels^9^. Synthesis of cGMP under control of cytoplasmic Ca^2+^ contributes to the restoration of the dark adapted state and mediates light adaptation of photoreceptors^1^,^2^,^3^.

Mammalian rod and cone cells express two forms of a sensory GC, assigned as GC-E and GC-F (alternatively named ROS-GC1 or 2, retGC1 or 2). Both GCs form homo-dimers in disc membranes of rod and cone outer segments, but recent research was mainly focused on GC-E, probably for the following reasons: (i) the fraction of total GC-E exceeds that of GC-F by 25-fold in bovine^10^ and 4-fold in mouse outer segments^11^; (ii) various retinal diseases like Leber´s congenital amourosis (LCA) and Cone-Rod-Dystrophies (CORD) correlate with mutations in the *GUCY2D* gene coding for photoreceptor GC-E^12^; (iii) mutations in the *GUCA1A* gene coding for GCAP1 correlate with cone, cone-rod and macular dystrophies in patients suffering from visual dysfunction, which is likely caused by an impaired operation of the GC-E/ GCAP1 complex, since GC-E is the preferred target of GCAP1^13^.

Deciphering the control mechanisms of cGMP synthesis in the GC-E/GCAP1 complex is important in understanding cGMP homeostasis in photoreceptor cells in health and disease. Previous work aimed at identifying the interaction sites of GCAP1 and GCAP2 in GC-E has led to inconsistent, even contradictory results^14^,^15^,^16^,^17^,^18^,^19^. While several studies indicated distinct binding sites for GCAP1 and GCAP2 localized in different regions in the cytoplasmic part of GC-E^14^,^15^,^16^,^19^, more recent studies suggested a differential activation mechanism in which GCAPs exchange at a shared single binding site upon fluctuating Ca^2+^ concentration^17^,^18^.

In the present study we investigated GC-E/GCAP interactions using a novel technique called Backscattering Interferometry (BSI), which allows label- and immobilization-free interaction analysis in physiologically relevant matrices at high sensitivity and in small volumes^20^,^21^,^22^. Using full-length human GC-E derived from heterologous expression in HEK 293 cells we quantified the binding of GCAP1 and GCAP2 to GC-E by BSI. It was also possible to test both of the currently proposed binding models: one, in which GCAP1 and GCAP2 interact at different sites in GC-E, and the other one where both NCS proteins share a single binding site. Control BSI assays were performed with the photoreceptor NCS protein recoverin that is known to bind to rhodopsin kinase GRK1 in a Ca^2+^-dependent manner^23^,^24^,^25^,^26^,^27^, enabling the determination of specific affinity in this complex physiological system.

## Results and Discussion

### Binding of Recoverin to GRK1

**Figure 1:**
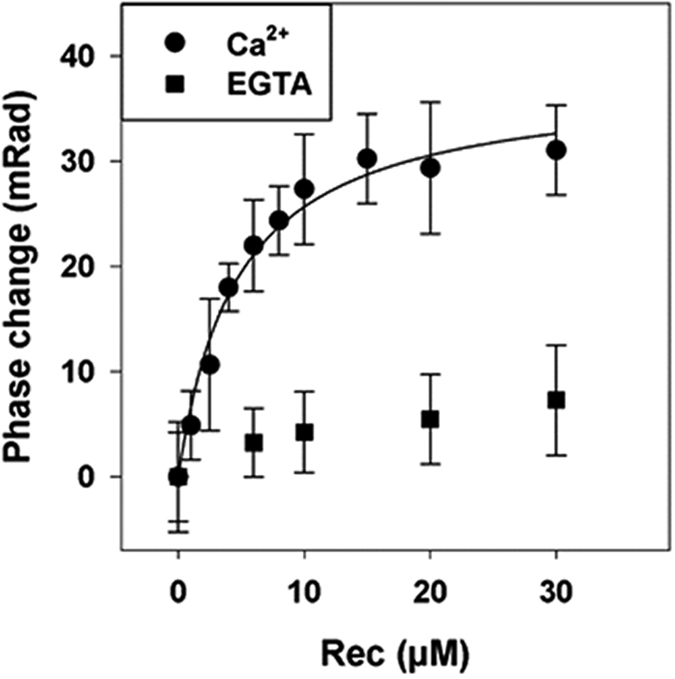
Interaction of recoverin (Rec) with the N-terminal GRK1 fragment (NRK) by BSI. A dilution series with increasing concentration of myristoylated bovine recoverin was prepared with a constant concentration of a GST fusion protein (4 μM) containing the *N*-terminal 25 amino acids of bovine GRK1. In the presence of 200 μM Ca^2+^ BSI reported a K_D_ of 4.72 μM ± 0.72 μM (number of replicates n = 4). In the absence of Ca^2+^ (EGTA) no binding signal was observed (n = 5). BSI data was fitted to a simple one site ligand binding model to obtain the K_D_ value.

BSI assays are relatively new and must be performed as relative measurements^21^. Therefore, we first employed recoverin as a test system for BSI performance benchmarking. Recoverin was chosen because, like GCAP1 and 2, it belongs to the family of NCS proteins and it exhibits similar three-dimensional folding^5^,^7^. It interacts with the *N*-terminal 25 amino acids of rhodopsin kinase (GRK1) thereby controlling its activity in a Ca^2+^-dependent manner. Here we quantified the binding affinity of recoverin to the N-terminal GRK1 fragment (NRK) employing the same GST fusion protein used earlier^24^. When increasing concentrations of recoverin were mixed with NRK at saturating Ca^2+^, we obtained a saturation isotherm yielding a K_D-_value of 4.72 μM ± 0.72 μM (Fig. 1). To test if the recorded phase change reflects specific binding of recoverin to NRK, we repeated the BSI assay in the absence of Ca^2+^. Figure 1 illustrates that, in the absence of Ca^2+^ no binding signal was detected. The K_D_-value for GRK1-recoverin interactions of 4.72 μM is similar to previous determinations of apparent binding constants, reported as 1 μM^25^, 10.7 μM^23^ and 41 μM^26^ using surface plasmon resonance (SPR) spectroscopy, 1.4 μM using isothermal titration calorimetry^27^ and 5.8 μM by titration of GRK1 inhibition^23^. SPR applications need immobilization of one interaction partner, which can create heterogeneous surfaces and might account for the larger variations observed with this technique. However, BSI enabled the quantification of GRK1-recoverin binding in free solution providing affinity constants that are not impacted by any modification such as immobilization. Further, the clear Ca^2+^-dependence of recoverin binding validates that BSI can be used to interrogate complex protein interactions.

### The binding of GCAPs to its target is Ca^2+^ independent

Although numerous studies covered the topic of localizing GCAP binding sites in photoreceptor GC-E in the last 20 years, the results are inconclusive^15^,^16^,^17^,^18^,^19^ in terms of defining the GCAP1/GC-E interaction interface. It is unclear as to why these experiments diverge, but the use of different experimental approaches might have led to contradicting results. Here, for the first time, we used a completely label-free experimental set-up to directly study the interaction of purified GCAP1 and GCAP2 with human GC-E.

**Figure 2:**
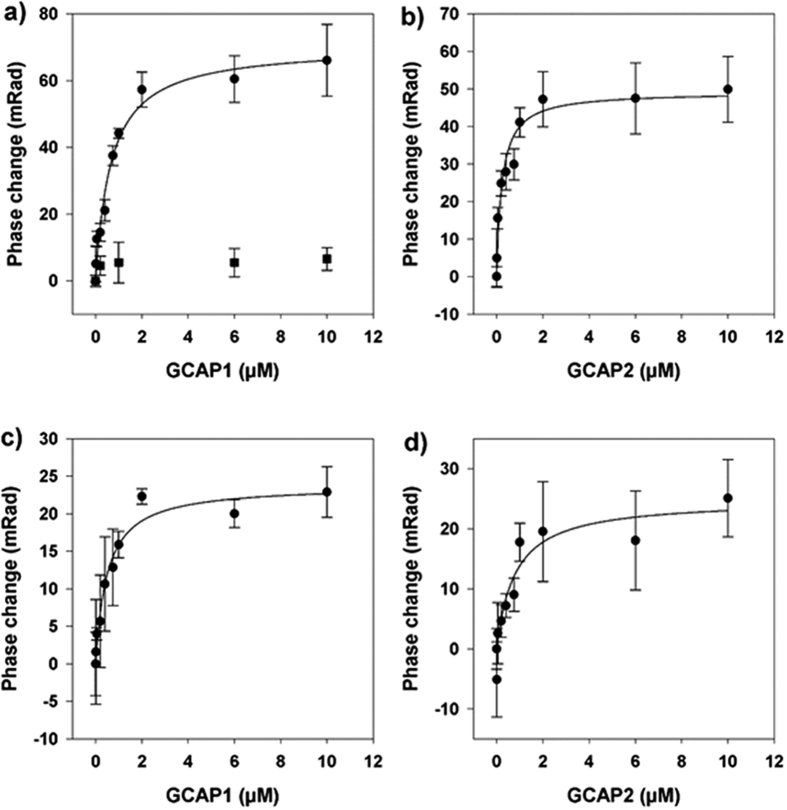
Interaction of GCAP1 and GCAP2 with the full-length human GC-E determined by BSI. In the top panel 1 mM EGTA was present, in the lower panel saturating 200 μM Ca^2+^ was present. The following K_D_ values were extracted from a fitting to a simple one ligand binding model: (**a**) GCAP1 in the presence of EGTA K_D_ = 663 nM ± 121 nM (**b**) GCAP2 in the presence of EGTA K_D_ = 231 nM ± 63 nM (**c**) GCAP1 in the presence of Ca^2+^ K_D_ = 505 nM ± 108 nM (**d**) GCAP2 in the presence of Ca^2+^ K_D_ = 418 nM ± 135 nM. Each data point is the average of 4 replications. The control titration displayed in (**a**) (■) of another EF-hand Ca^2+^-binding protein, calmodulin (CaM-S17C) resulted in a minor phase shift, showing that the interaction between human GC-E and the GCAPs is a specific binding event. Concentration of calmodulin was 0, 0.75 μM, 6 μM and 10 μM.

HEK cell membrane vesicles containing full-length active human GC-E with an average vesicle size distribution of around 50 nm were titrated with increasing GCAP concentrations. Phase changes from BSI recordings which signal chemical interactions were performed in the absence of Ca^2+^ and saturated between 5–10 μM of GCAP1 or GCAP2 (Fig. 2a,b) resulting in halfmaximal apparent K_D_-values of 663 ± 121 nM and 231 ± 63 nM, respectively. Both GCAP forms bind in the submicromolar range to the target GC in agreement with previously performed enzyme activation assays using a native GC-E source^28^,^29^,^30^. However, due to the inhibition of mammalian GC-E by GCAPs in the presence of Ca^2+^, apparent affinity constants (EC_50_) from enzyme activation assays are difficult to obtain and prone to large experimental errors. Yet, this observation is not a limitation for a BSI assay.

When performing the BSI titration in the presence of Ca^2+^ (Fig. 2c,d) we quantified affinity constants for GCAP1 (K_D_ = 505 nM ± 108 nM) and GCAP2 (K_D_ = 418 nM ± 135), which are similar values to those determined in Ca^2+^-free solution. GCAP2 did show a slightly higher affinity for human GC-E in the apo- and Ca^2+^-bound state than did the GCAP1 species, but within experimental error both of the K_D_-values were found to be in the submicromolar range. By using the BSI assay approach, it was possible to confirm previous circumstantial evidence reporting Ca^2+^-independent interaction of GCAPs with mammalian GCs^29^. Furthermore, we showed that the binding process is of medium affinity, which is similar to previous reports of EC_50_-values. Concentrations of GCAPs, at which the activation of GCs is halfmaximal are expressed as EC_50_-values and are interpreted as apparent affinity constants^11^.

The validity of these observations were confirmed by excluding impairment functionality of human GC-E in HEK vesicles, by performing a standard GC-Assay^31^ and confirming enzymatic functionality (data not shown). In this case, HEK cell membranes expressing human GC-E were processed using sonication to form vesicles^20^. Reference recordings were made by BSI with HEK cell vesicles lacking human GC-E. Additionally we used calmodulin (CaM), another member of the EF-hand superfamily of Ca^2+^-binding proteins, which is very similar to NCS proteins in three dimensional folding^5^,^32^, to test whether the BSI binding signal is specific for GCAPs. The result of this binding assay is depicted in Fig. 2a, showing that titration of GC-E with increasing concentration of calmodulin resulted in a phase shift below the limit of detection for binding. This result further confirms that the BSI signal is quantifying specific binding of the GCAPs to human GC-E (Fig. 2).

### The juxta-membrane and kinase homology domain exclusively interacts with GCAP1

**Figure 3:**
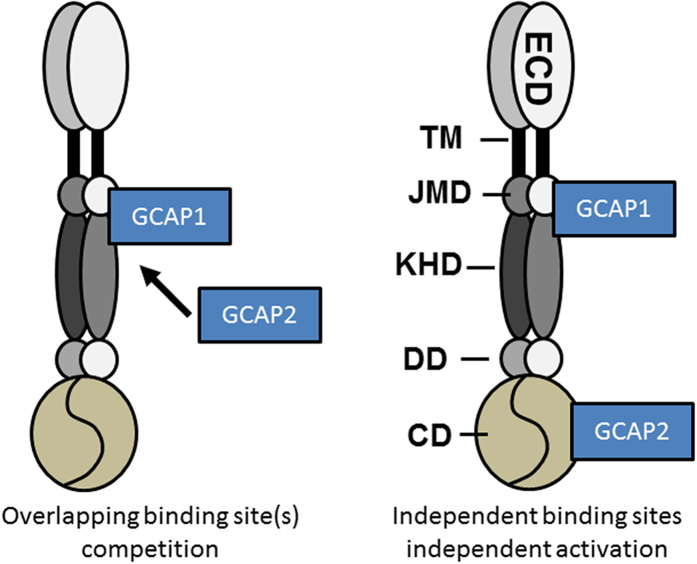
Model of interaction between mammalian GC-E, GCAP1 and GCAP2. Domains of GC-E are assigned as extracellular domain (ECD, corresponding to the intradiskal part in rod disk membranes), transmembrane domain (TM), juxta-membrane domain (JMD), kinase homology domain (KHD), dimerization domain (DD) and catalytic domain (CD). Two contradicting concepts of GCAP binding to GC-E are shown. References and details are provided in the main text. For reasons of simplicity, we sketched the GC/GCAP complex showing only one GC dimer. Each GCAP form might also bind separately to a GC dimer.

Having proven the suitability of the BSI assay system for the GC/GCAP system we set out to critically test opposing opinions about the localization site of GCAP1 and GCAP2 interaction domains. This task was uniquely enabled by using BSI to study the molecular interactions of intracellular parts of human GC-E. Here we divided GC-E into two fragments, one comprising the amino acid residues M496 to K806 corresponding to the juxta-membrane and kinase homology domain (JMD + KHD)^16^. The other fragment corresponded to the catalytic domain (CD) found in the region from G868-S1103^14^. Both of these fragments were fused to a solubility enhancer, maltose binding protein (MBP) to ensure they were soluble. Either GCAP1 or GCAP2 would bind to both fragments or both GCAPs would target to different regions. The topology of a G-E dimer is shown in Fig. 3 indicating protein domains and the two alternative scenarios of GCAP interaction that are currently discussed.

**Figure 4:**
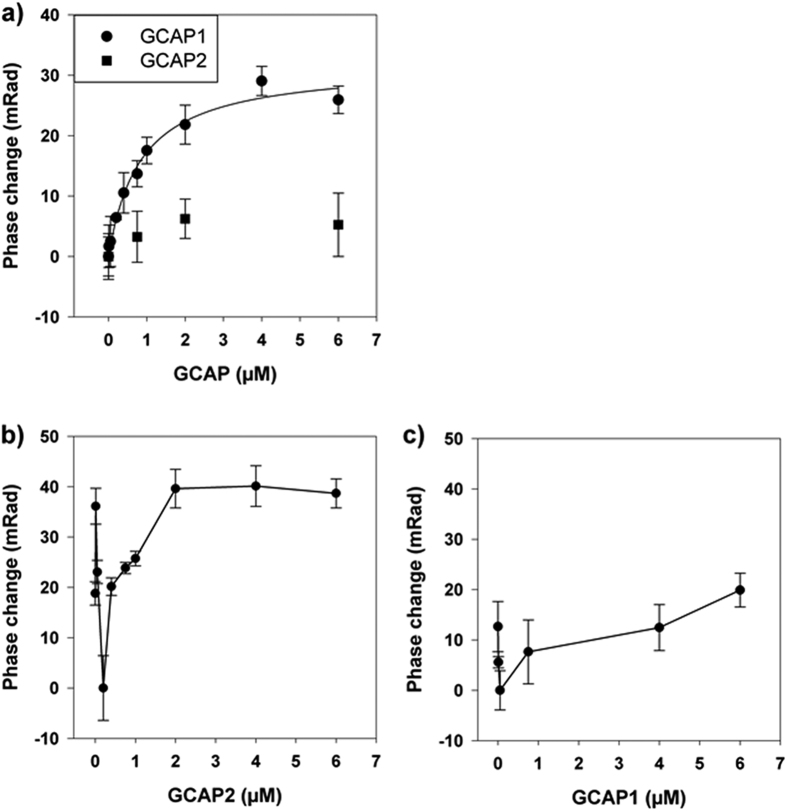
Interaction of GCAP1 and GCAP2 with fragments of human GC-E determined by BSI in the presence of 200 μM Ca^2+^. (**a**) The Interaction of GCAP1 and GCAP2 was tested with the JMD + KHD fragment. An Interaction of GCAP2 with this fragment was not observed, for GCAP1 the fitting of BSI data gave a K_D_ = 841 nM ± 134 nM (n = 3). (**b**,**c**) Interaction of GCAP2 and GCAP1 with the CD fragment of human GC-E (n = 4).

Figure 4a presents the results from the BSI assay performed with GCAP1 and GCAP2 on the JMD-KHD fragment. Importantly, binding of GCAP1 to the JMD-KHD fragment was observed, with a K_D_-value of 841 nM ± 134 nM, while GCAP2 showed only a minor BSI signal (phase shift). The lower curve in Fig. 4 clearly indicates this signal is not of the magnitude to be considered a quantifiable binding event. Further, since all reference measurements were performed with the exact same concentration of MBP and subtracted from the BSI binding signal we can exclude a possible interaction of the fusion MBP part with the GCAPs. Taking into account that saturating Ca^2+^ was present in the measuring buffer, which might have affected the binding of GCAP2 to the JMD + KHD fragment, we reproduced the same BSI assay except 1 mM EGTA was present (data not shown). The Ca^2+^-bound state of either GCAP1 or GCAP2 had virtually no influence on the interaction with the JMD-KHD fragment. To further validate our observations we used isothermal titration calorimetry (ITC) and titrated either GCAP1 or GCAP2 with the JMD + KHD fragment at 25 °C. Using a one binding site model implemented in the ITC evaluation software (Origin 4.0, MicroCal) we determined an affinity constant for GCAP1 binding that is consistent with the BSI data (K_D_ = 580 nM ± 250 nM) (data not shown). The stoichiometry of the binding shows a one to one binding for GCAP1 to the GC-fragment (n = 0.9 ± 0.2). However, we were only able to record two ITC runs of sufficient quality, four different runs showed no detectable enthalpy change. We attribute these findings to the inherent instability of the JMD + KHD fragment and consider the BSI assay as advantageous over ITC investigating for targets, which are intractable. In the experimental set-up of our ITC measurements, the JMD + KHD fragment had to be placed in the measuring chamber for several hours at 25 °C, whereas for the BSI assay the JMD + KHD sample is kept on ice shortly before the injection started.

Results from the binding experiments for the GCAPs to the CD fragment are more difficult to interpret: amplitudes of GCAP2 binding titration showed a dip at very low GCAP2 concentrations, but then displayed increasing amplitudes saturating at 40 mRad (Fig. 4b). Binding signals of GCAP1 were lower and data showed higher scatter or poorer reproducibility (Fig. 4c). These data are consistent with previous reports showing interaction of GCAP2 with the CD domain and interaction of GCAP1 with lower affinity^15^. However, we can only speculate where the initial BSI signal decrease upon GCAP2 titration to the CD-fragment came from. Membrane bound GCs, either hormone ligand receptors or sensory GCs form dimers as functional units^33^,^34^. Adding GCAP2 to the CD-fragments might have induced a dimerization of the fragment, which is reflected in the negative BSI signal relative to observations reported above. This observation would be consistent with a previous report showing that the presence of GCAPs facilitates formation of dimers in photoreceptor GC-E^35^ and with a recent report on the BSI signaling mechanism that shows certain interactions can produce a negative-going binding curve^21^.

### GCAPs do not share a single binding site

Recently Peshenko *et al*.^17^ identified a crucial amino acid residue (M823) located in the dimerization domain of human GC-E involved in GCAP binding by a co-localization approach. Further the same group suggested a model in which GCAP1 and GCAP2 share a single binding site located in a region spanning the juxta-membrane domain to the dimerization domain, shown by a comprehensive study employing chimeras of human GC-E and its co-localization with GCAPs^18^. Such a scenario, in which GCAP1 and GCAP2 interact with GC-E in a mutually exclusive mode upon fluctuating Ca^2+^- levels is at odds with our findings here and with several other reports^14^,^15^,^16^,^19^. Here we quantified affinities for GCAPs by BSI in the sub-micromolar range, which were negligibly affected by the Ca^2+^-bound state of GCAPs. Bovine rod outer segments contain a GCAP1 and GCAP2 concentration of about 3 μM^28^, which would result in the GC-E being saturated, assuming K_D_ values determined in this study. However, the medium affinity binding of GCAPs to full-length GC-E could also reflect a dynamic complex formation and dissociation, which occurs in the absence and presence of Ca^2+^. The GC-E/GCAP complex might undergo conformational rearrangements that could be the reason why different experimental approaches (peptide competition, mutagenesis and crosslinking in combination with mass spectrometry) gave different, in some cases inconsistent results.

## Conclusion

The BSI system was used in the last decade in protein interaction studies^22^,^36^,^37^,^38^,^39^,^40^,^41^,^42^, and the clear Ca^2+^-dependent interaction of recoverin with its target GRK1^23^,^24^,^25^,^26^,^27^ represents a system in which we can induce binding simply by adding low concentrations of Ca^2+^, clearly showing that a BSI binding signal is specific. The signal occurs only in the presence of Ca^2+^, whereas no binding signal was recorded in the absence of Ca^2+^. BSI enabled the resolution of contradicting findings regarding GCAP interaction sites and we are able to report that GCAP1 and GCAP2 do not share a common binding site. Planned investigations for future studies beyond the scope of this work will address the investigation of more complex BSI response patterns. These might result from a shift in protein monomer-dimer equilibria producing negative going signals.

## Methods

### Molecular cloning of GC-fragments

The cloning of full length human GC-E into a pIRES2-eGFP vector (Clontech) for the expression in mammalian cell culture was described earlier^31^.

Without a fusion part we were not able to obtain any soluble fragment from overexpression in *E. coli*, due to the strong tendency of constructs to aggregate. Hence, we fused the respective fragments to the solubility enhancer maltose binding protein (MBP), which allowed to obtain a soluble fragment after dialytic refolding from the inclusion bodies. Fusion proteins were designed accordingly and named MBP-KHD and MBP-CD. The MBP-KHD is a fusion protein with maltose binding protein (MBP) attached to a human GC-E fragment corresponding to the juxta-membrane and kinase homology domain (JMD-KHD, M496-K806). MBP-CD corresponds to the catalytic domain (CD, G868-S1103) of the human GC-E. The coding sequence of KHD was amplified on wildtype human GC-E sequence by PCR adding a BamHI and a partial NcoI site using the primers 5′-GTCTCCGGCCCCAACAAG-3′ and 5′-AAAGGATCCTCACTTGAACAGGTCGAAGGTGTG-3′. The M11-MBP vector (provided by the EMBL, Heidelberg, Germany)^43^ was cut by NcoI, treated by Klenow fragment to obtain a blunt end site and afterwards cut by BamHI. The PCR fragment was cut by BamHI, phosphorylated with T4 Polynucleotide Kinase and ligated into the M11-MBP using standard protocols. MBP-CD was obtained by PCR on wildtype sequence by PCR adding restriction sites BamHI and NcoI (5′-AAAACCATGGGGACACCAGTGGAGCCCG-3′, 5′-AAAGGATCCTCAAGAGAACTGGCCCGGCCG-3′) and ligated into M11-MBP vector (EMBL) following standard protocols. Sequences of the fragments were verified by DNA sequencing (GATC, Germany).

### Protein expression and purification

MBP constructs were expressed in *E.coli* BL21 (+) cells. For this *E. coli* cells were transformed with the respective plasmid and grown to an OD_600_ = 0.6. Expression was induced by 1 mM IPTG (isopropyl β-D-1-thiogalactopyranoside), after 4 h the cells were harvested at 5000 × g for 15 min and the pellet was resuspended in 20 mM HEPES KOH pH: 7.4, 1 mM DTT, 1 mM PMSF, 5 U/mL DNAse and lysed by sonification on ice. To separate insoluble from soluble proteins the lyzed cells were centrifuged at 50,000× g for 1.5 h at 4 °C. The MBP constructs were exclusively present in the insoluble inclusion bodies verified by SDS-PAGE. To extract the constructs the insoluble pellet was washed twice with 20 mL of 20 mM HEPES/KOH pH 7.4 and homogenized in 30 mL 8 M Urea and incubated overnight at 4 °C. After centrifugation at 50,000 × g for 30 min at 4 °C the MBP construct containing supernatant was dialyzed twice against 3 L of 20 mM Tris/HCL pH 7.5, 150 mL NaCl, 1 mM DTT for at least 4 h and centrifuged again at 50,000 × g for 30 min at 4 °C. To isolate the respective MBP construct the supernatant was loaded onto approximately 10 mL of amylose resin (NEB) in a self-packed gravity flow column at 4 °C. After washing with 10 column volume 20 mM Tris/HCL pH 7.5, 150 mL NaCl, 1 mM DTT the MBP constructs were eluted using the same buffer supplemented with 10 mM maltose. The buffer was exchanged to 50 mM (NH_4_)HCO_3_ and protein sample were lyophilized and stored at −80 °C until further use.

For reference measurements MBP was obtained by cleaving the respective GC fragments with TEV-Protease. For this, the MBP-GC construct was dissolved in 20 mM Tris/HCL pH 7.5, 150 mM NaCl, 1 mM DTT and TEV protease was added in a ratio of 1:10 and incubated overnight at 4 °C. The purification of the MBP fragment was done exactly as described for the MBP-GC constructs, except that susbsequently a size exclusion chromatography was performed using 20 mM Tris/HCl pH 7.5, 150 mL NaCl, 1 mM DTT and a HiLoad 26/60 Superdex 75 prep grade gel filtration column (GE Healthcare).

GCAP1 and GCAP2 was expressed in *E. coli* and purified by a combination of size-exclusion chromatography and anion-exchange chromatography exactly as described before^28^,^44^. To obtain myristoylated GCAPs *E. coli* cells were co-transformed with yeast *N*-terminal myristoyl transferase (kindly provided by Dr. Jeffrey Gordon, Washington University School of Medicine, St. Louis, USA) and the LB medium was supplemented with myristic acid. Myristoylated Recoverin and the *N*-terminal GRK1 fragments (NRK) was prepared exactly as described earlier^23^,^24^.

Human GC-E was expressed in HEK-flip 293 cells as described previously^31^,^45^. Briefly, HEK cells were cultivated in minimal essential medium to 80% confluency and harvested in 5 mM KCl, 20 mM MgCl_2_ and 150 mM phosphate buffer (3–5 × 10^6^ cells). 5 μg Vector DNA (pIRES2-eGFP, *Clontech*) containing the cDNA for human GC-E was added and electroporated with the CLB system (Lonza). After 24 h the medium was exchanged and supplemented with G418 (Merck Millipore), a selective antibiotic to generate a stable cell line expressing human GC-E. Single colonies of HEK cells were picked and checked by western blot for the expression of human GC-E. All experiments were done using a stable cell line originating from a single cell. For the experiments the stable cell line was cultivated up to a confluency up to 80% and harvested (500xg, 5 min) and washed twice with ice-cold PBS to remove medium residues. All cell pellets were frozen in liquid nitrogen and stored at −80 °C until further use.

### Isothermal titration calorimetry

Isothermal titration calorimetry (ITC) was performed using a VP-ITC instrument from MicroCal (Northampton, MA, USA) at T = 25 °C. Purified MBP fusion protein was present in the recording cell in the exact same buffer used for the BSI assays (30 mM Mops/KOH pH 7.2, 60 mM KCl, 4 mM NaCl, 1 mM DTT, 3.5 mM MgCl_2_, 1 mM GTP, 0.3 mM ATP) at 0.5 μM and titrated with myristolated GCAP1. All protein samples were dissolved in filtered (0.22 μm) and degassed titration buffer and sonicated at 0 °C for 5 min. Afterwards the protein samples were ultracentrifuged (100.000 × g, 15 min) and the supernatant was carefully withdrawn for the subsequent ITC experiments.

GCAP was placed in the microsyringe chamber at 10 μM and the titration was performed by subsequent injections of 5 μl GCAP solution into the recording cell keeping a time interval of 180 s between injections and an initial delay of 600 s after temperature equilibration. Control injections of GCAP into MBP, lacking a GC-E fusion part were also performed accordingly and subtracted from the sample titration. The data was fitted using a one ligand binding model employing the Microcal software (Origin) to estimate a binding constant (K_D_) at the given temperature.

### BSI instrumentation

BSI instrumentation has been described in detail previously^22^,^46^,^47^. Briefly, the instrument is comprised of a linear polarized helium–neon laser (2 mW, Melles Griot) a microfluidic chip (Micronite, Netherlands), and a charge-coupled device (CCD) camera (CCD-S3600-D, Alphalas, Germany). The respective sample is introduced into the microfluidic chip, which is designed to create a resonance cavity with a long effective path length. The incident coherent light is converted into an interferometric fringe pattern, which is captured by the CCD camera. Fourier analysis is used to determine the phase change (in radians) to quantify spatial position changes of the fringes due the binding of a protein, which induces a change in refractivity. The change in refractive index was shown to correlate with protein-protein binding^21^,^22^ and are used to obtain equilibrium dissociation constants (K_D_). All BSI assays were performed using a BSI setup in the Bornhop Group (Vanderbilt University, USA), except the experiments employing Rec-GRK1, which was done in the Koch Group (University of Oldenburg, Germany) with a BSI setup identical to the one described afore.

### Protein interaction assay using BSI

The interaction studies were performed using either full-length human GC-E or fragments of human GC-E expressed as MBP fusion proteins. For the BSI interaction studies with full-length human GC-E, HEK vesicles expressing GC-E were prepared as follows. Approximately 4 × 10^6^ cells were resuspended in titration buffer (30 mM Mops/KOH pH 7.2, 60 mM KCl, 4 mM NaCl, 1 mM DTT, 3.5 mM MgCl_2_, 1 mM GTP, 0.3 mM ATP) supplemented with 1 mM PMSF and probe sonicated for 2 min. The particle size of the resulting HEK cell vesicles was determined by dynamic light scattering to range around 50 nm. Afterwards the total protein amount was determined using a standard Bradford assay^48^ and the HEK vesicles were diluted with titration buffer to a final concentration of 50 μg/mL. As a reference Mock-HEK cells that do not express human GC-E were treated accordingly. The analyte GCAP1 was dissolved in the titration buffer and a dilution series ranging from 0–20 μM was prepared. Afterwards equal volumes of either GC-HEK vesicles or Mock-HEK vesicles and GCAP were mixed to obtain a constant concentration of HEK vesicles and increasing GCAP concentration ranging from 0–10 μM. Protein samples were allowed to reach equilibrium for at least 1 h at 4 °C and were brought to room temperature for one additional hour. The BSI assay employing the respective GC-fragment were done accordingly. A dilution series with myristoylated GCAP was prepared ranging from 0 to 6 μM with a constant concentration (375 nM) of the respective MBP fusion protein. As a reference we used MBP lacking a GC-E fragment.

For the assay of recoverin and GRK1 we used buffer condition (20 mM HEPES/KOH pH 7.5, 100 mM NaCl, 1 mM MgCl_2_, 200 μM CaCl_2_ or 1 mM EGTA) to match previously SPR studies employing this system^24^. Since the *N*-terminal GRK1 fragment has a GST fusion part, we used GST alone in a reference dilution series.

## Additional Information

**How to cite this article:** Sulmann, S. *et al*. Label-free quantification of calcium-sensor targeting to photoreceptor guanylate cyclase and rhodopsin kinase by backscattering interferometry. *Sci. Rep.* **7**, 45515; doi: 10.1038/srep45515 (2017).

**Publisher's note:** Springer Nature remains neutral with regard to jurisdictional claims in published maps and institutional affiliations.
